# Supplementary material for: Systematic identification of metabolites controlling gene expression in E. coli
Source: Nat Commun. 2019 Oct 2;10:4463. doi: 10.1038/s41467-019-12474-1 (PMC6775132; doi:10.1038/s41467-019-12474-1)
Supplement: Supplementary file 1 — Supplementary Information [file 41467_2019_12474_MOESM1_ESM.pdf]

Supplementary Information

Systematic identification of metabolites controlling gene expression in *E. coli*

Lempp *et al*.

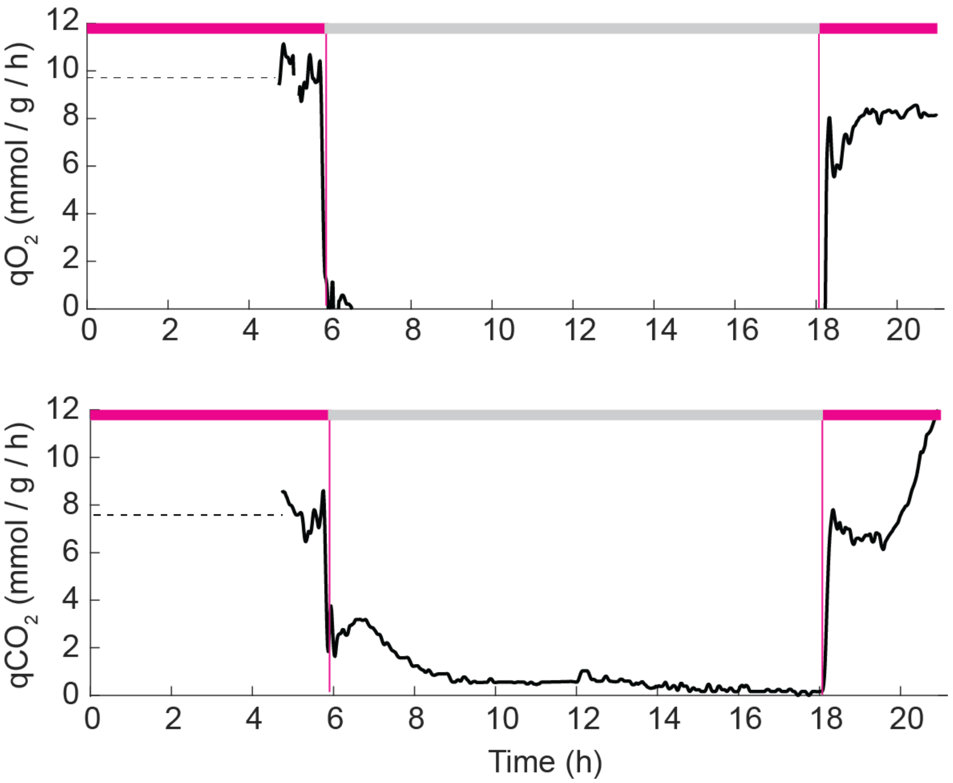

Supplementary Figure 1. Specific oxygen consumption (qO_2) and carbon production rates (qCO_2) during the growth-starvation-growth switch. The dashed line indicates the phase when biomass was too low to determine rates. Pink indicates growth phases and grey the starvation phase. (Source data are provided as a Source Data file.)

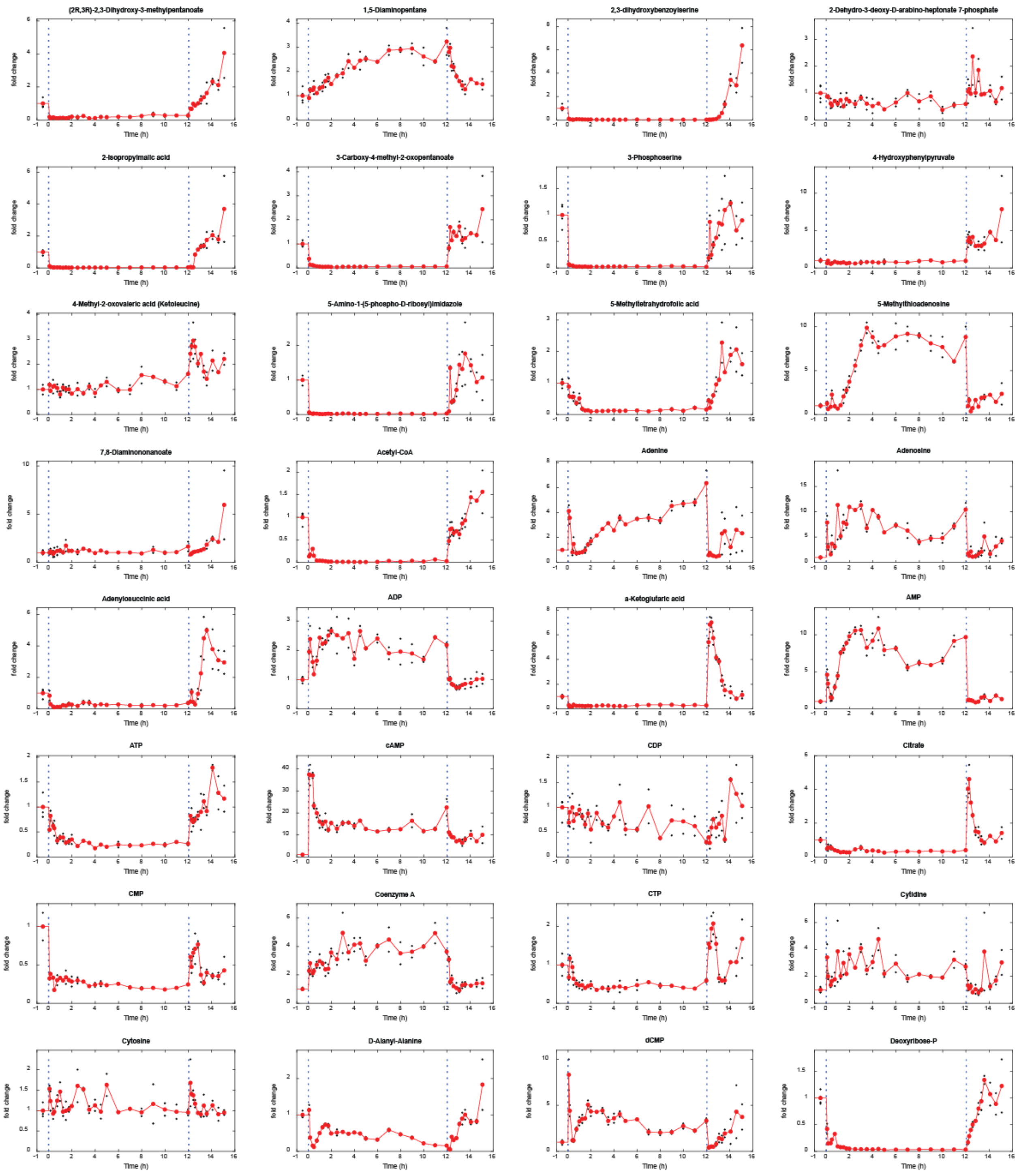

Supplementary Figure 2. Metabolite levels during the switch between starvation and growth. Relative concentrations are shown as fold change relative to the first data point. Black dots show levels of two replicates per time point (four at the first time point), red dots are the mean. Dashed vertical lines indicate the growth-starvation-growth switch.

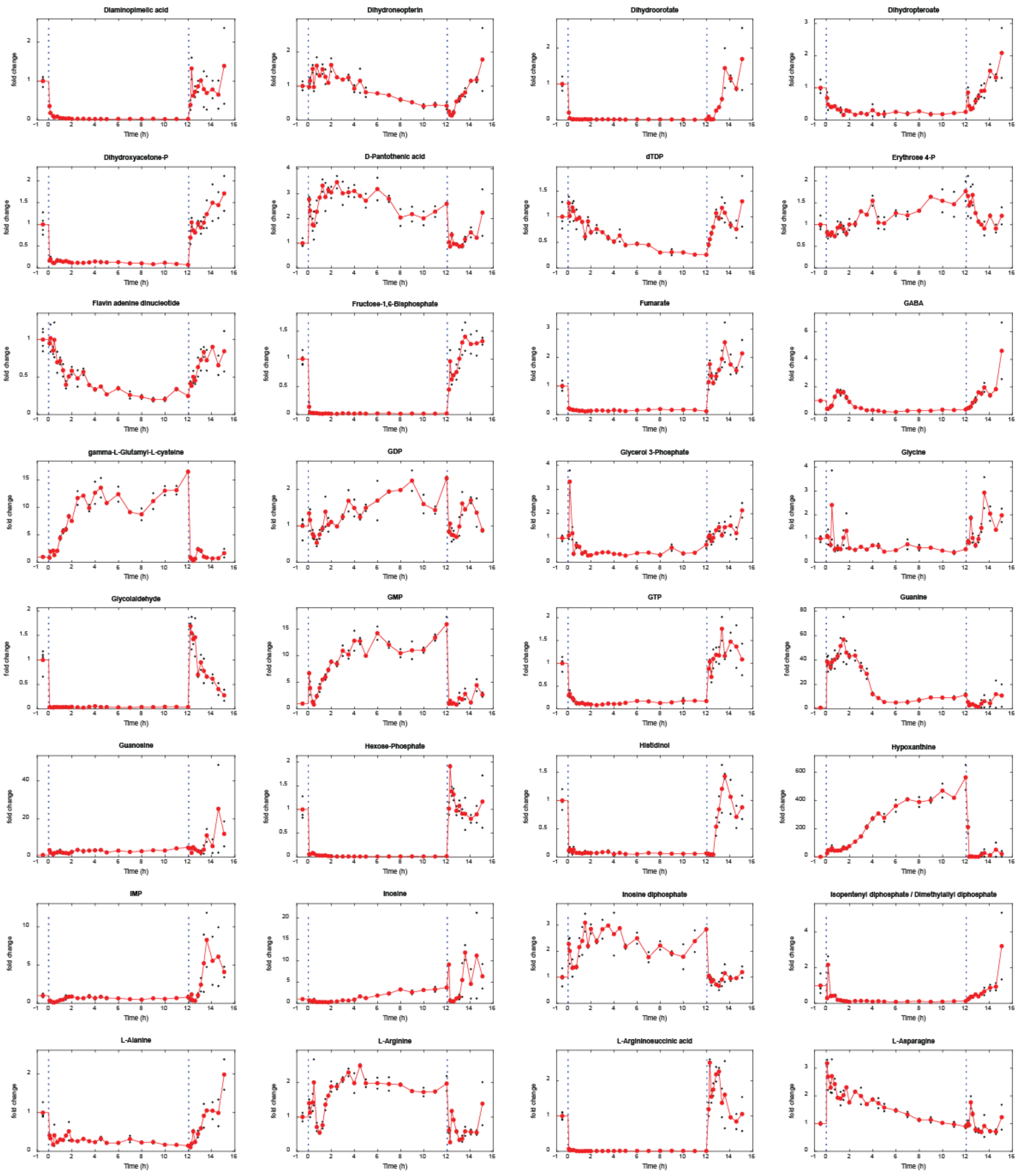

Supplementary Figure 2. (continued)

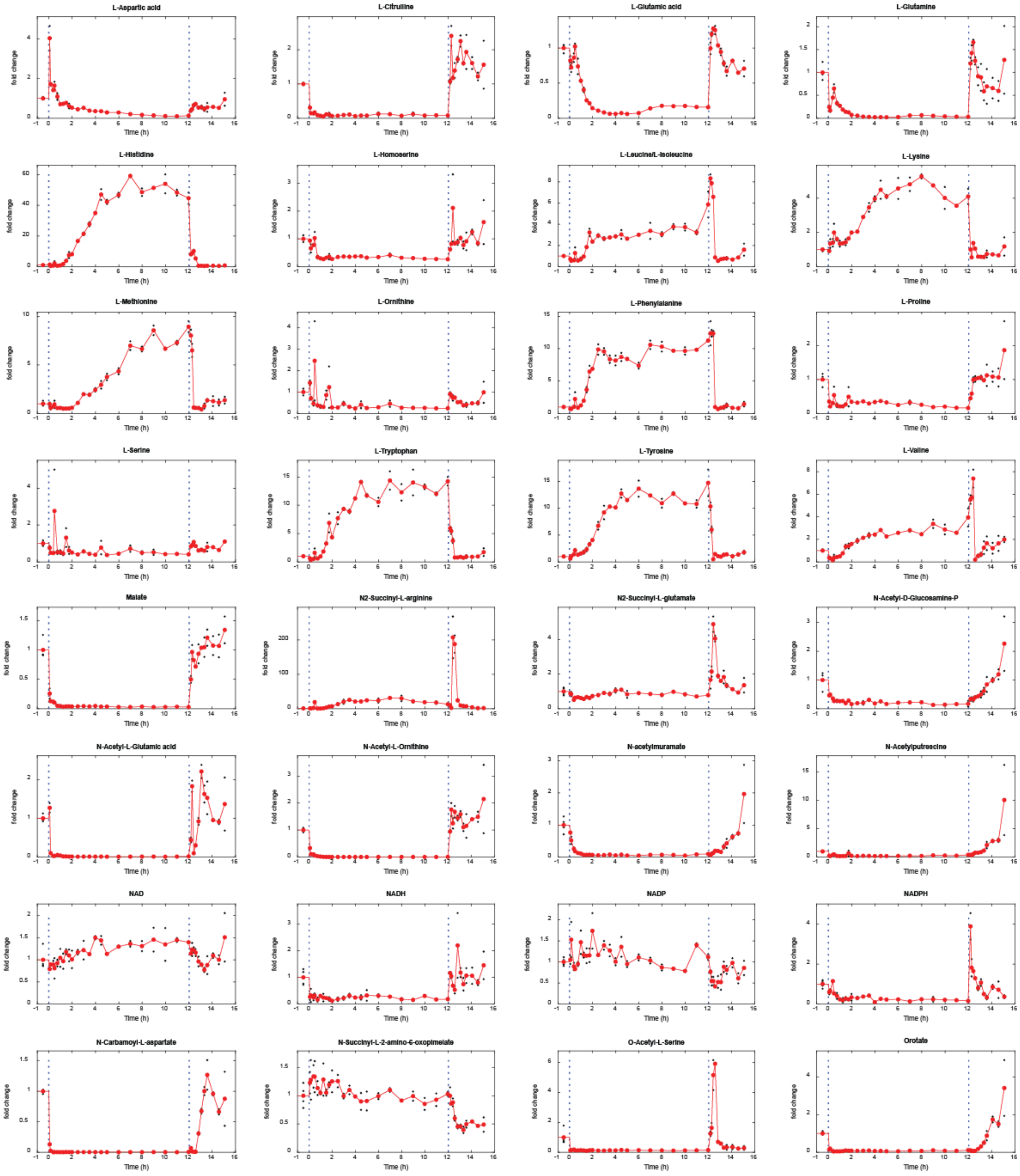

Supplementary Figure 2. (continued)

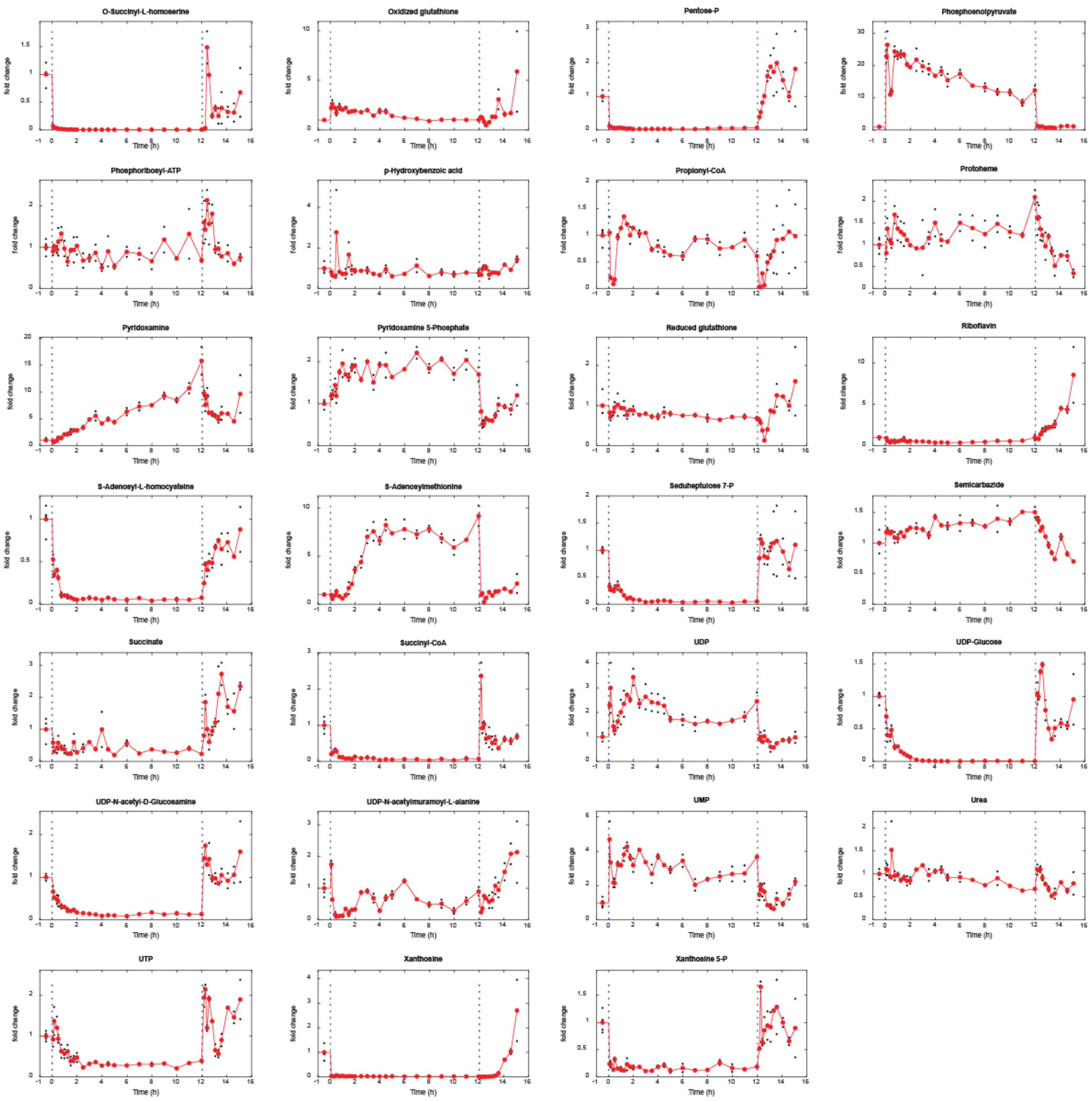

Supplementary Figure 2. (continued)

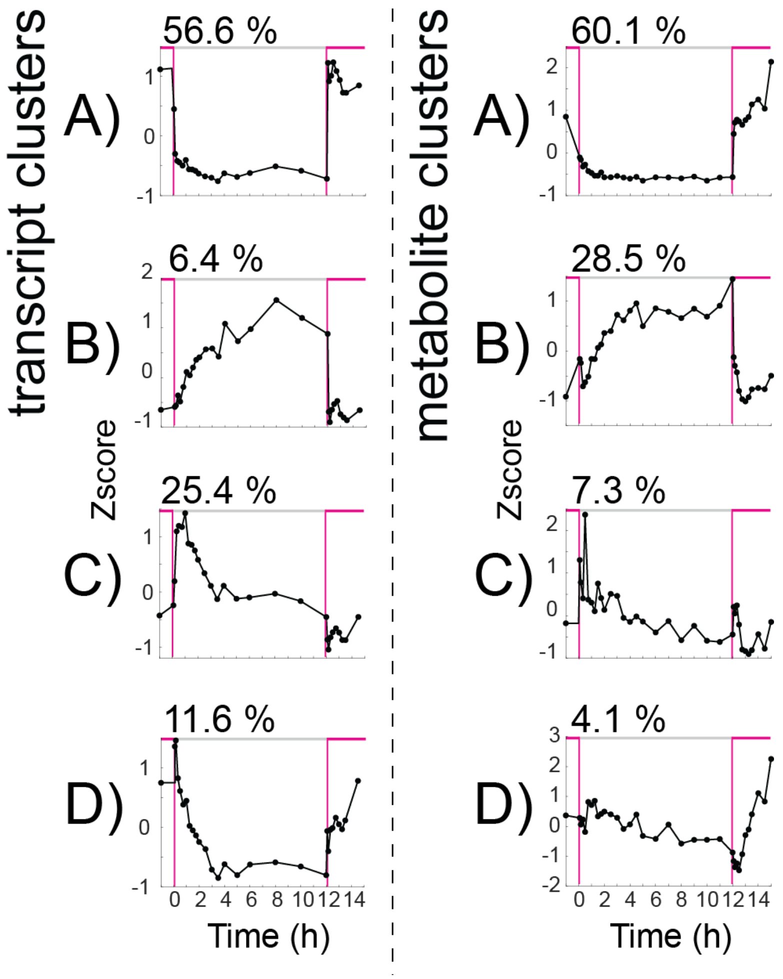

Supplementary Figure 3. Average dynamics of the hierarchical clusters A - D of transcripts and metabolites. Percentages show the number of metabolites/transcripts in cluster relative to the total number of measured metabolites/ transcripts. Pink indicates growth phases and grey the starvation phase. (Source data are provided as a Source Data file.)

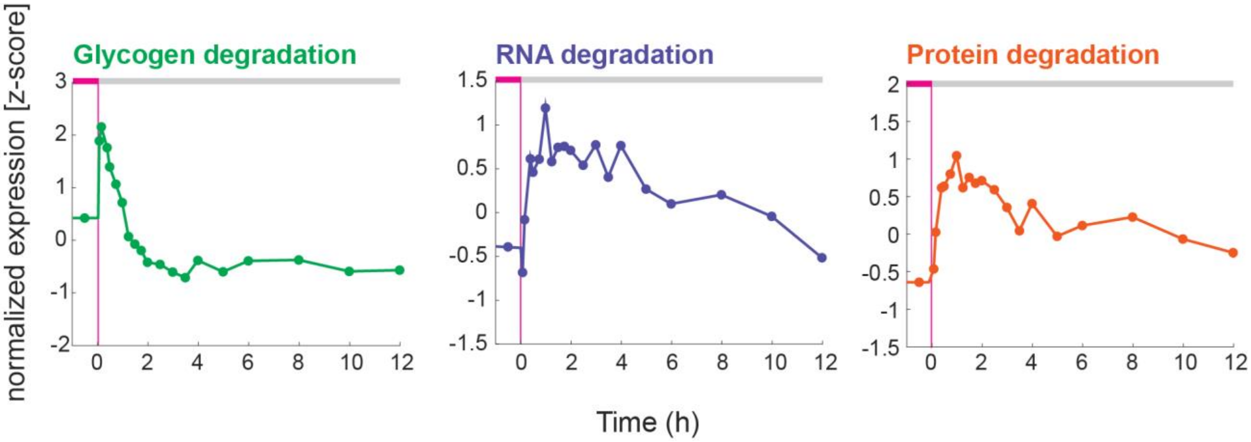

Supplementary Figure 4. Average expression of genes in degradation pathways of glycogen (green), RNA (purple) and proteins (orange) during the starvation phase. Pink indicates the growth phase and grey the starvation phase. Expression profiles include only genes that increase during the starvation phase (Glycogen degradation: 4 of 6 genes; RNA degradation: 13 of 35 genes; Protein degradation: 42 of 106 genes) (Source data are provided as a Source Data file.)

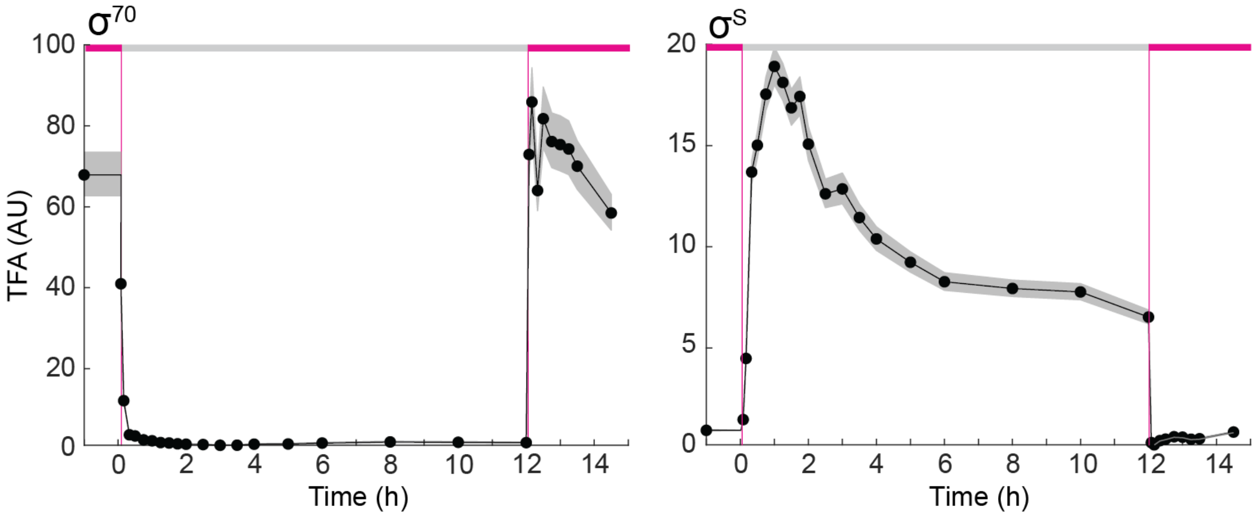

Supplementary Figure 5. Activity of the sigma factors σ^{70} and σ^{S} during the switch between starvation and growth. The grey area indicates the 95% confidence interval of n = 100 randomized estimations with Network Component Analysis. Pink indicates growth phases and grey the starvation phase. (Source data are provided as a Source Data file.)

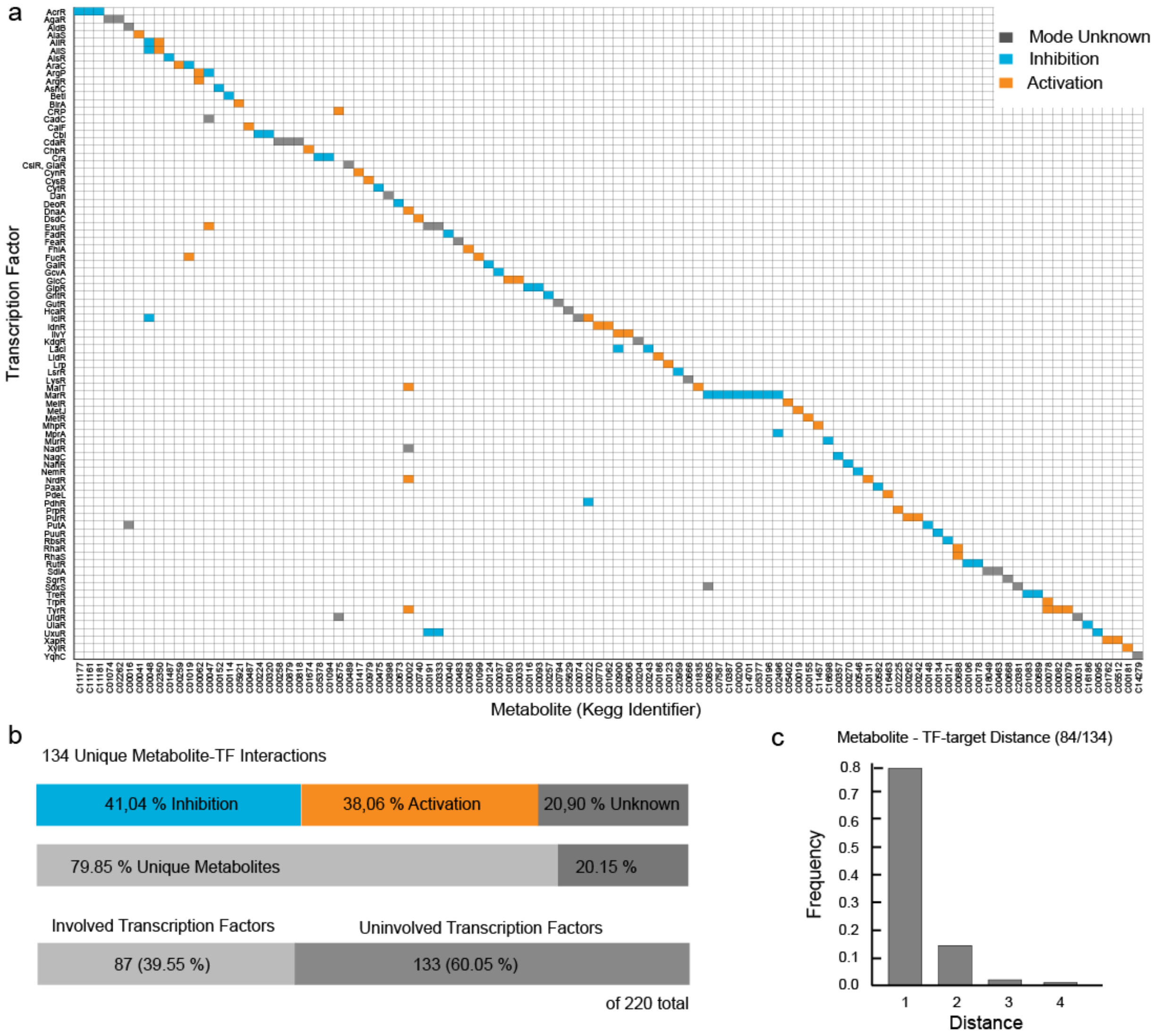

Supplementary Figure 6. a) Metabolite-transcription factor interactions that are described in the literature and databases. Shown are 87 transcription factors (rows) and their respective effector metabolites (columns). Orange indicates an activation of the TF by the metabolite, blue indicates an inhibition, and grey indicates that the mode is unknown. **b)** Mode of metabolite-transcription factor interactions; fraction of unique metabolites in the 134 different interactions; number of transcription factors for which an interacting metabolite is known. **c)** Distance between a metabolite and the target genes of the interacting transcription factor. The distance d was transformed by the following equation to account only for genes: Distance=(d+1)/2.

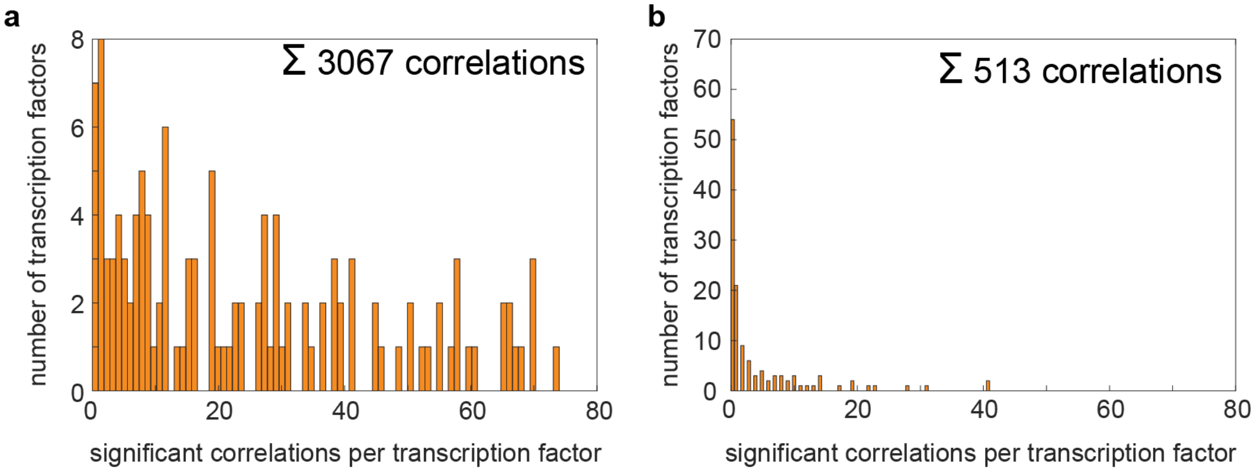

Supplementary Figure 7. a) Number of significant kinetic correlations (R^2>0.75) per transcription factor without distance filter. **b)** Number of significant kinetic correlations (R^2>0.75) per transcription factor with the distance filter. (Source data are provided as a Source Data file.)

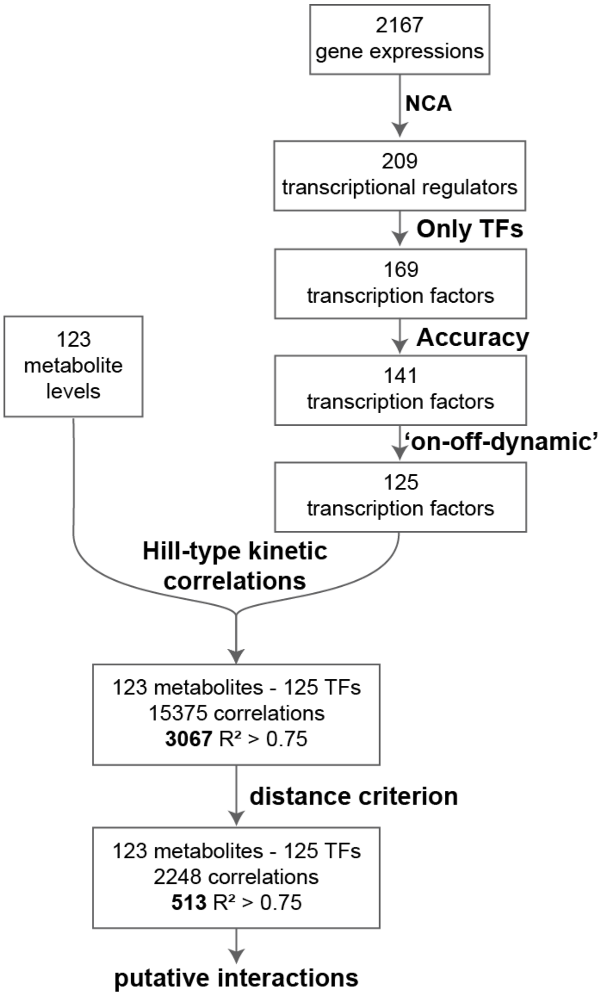

Supplementary Figure 8. Steps to reduce the number of correlating metabolite-transcription factor pairs.

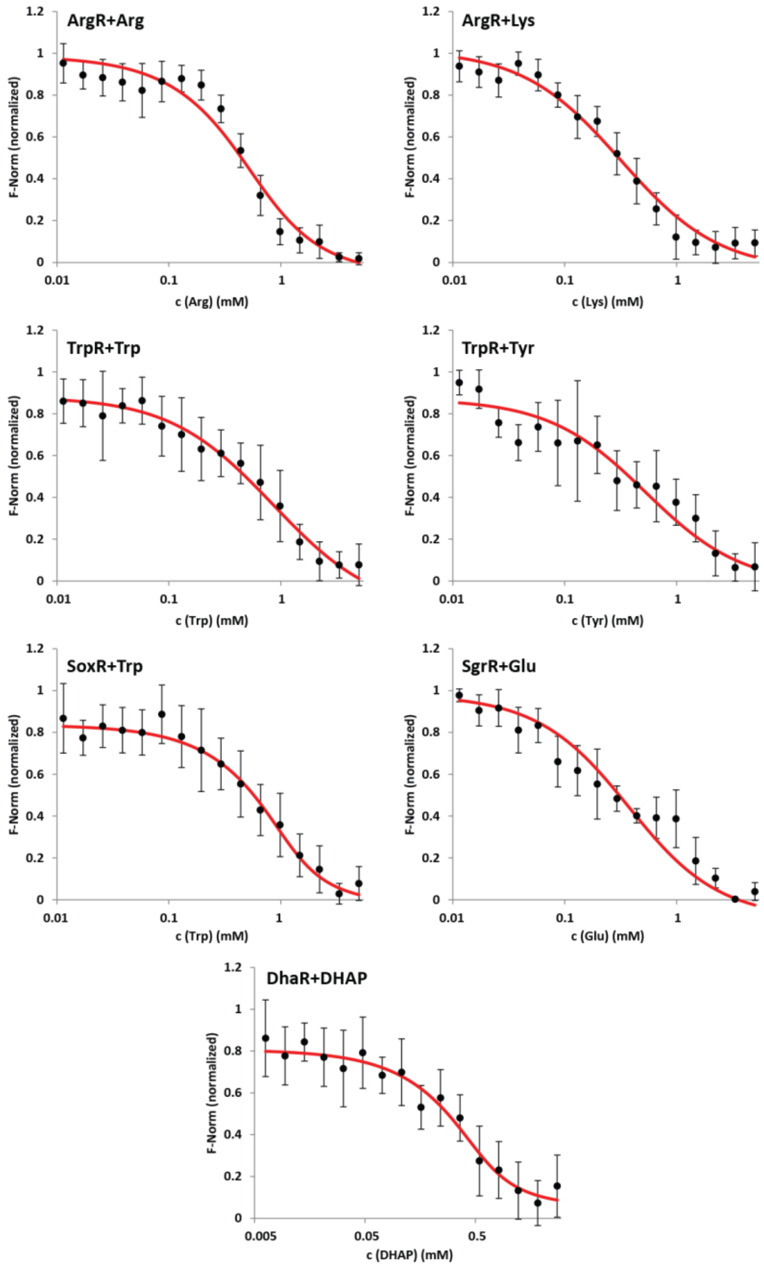

Supplementary Figure 9. *In vitro* binding assays with micro-scale thermophoresis (MST). Normalized fluorescence (black dots) was fitted (solid red line) to calculate the binding affinity of each interaction. The respective transcription factor (50 nm) was titrated with increasing amount of the putative effector metabolite. Shown are the mean and standard deviation of n = 9 MST assays (three technical replicates of three biological replicates). (Source data are provided as a Source Data file.)

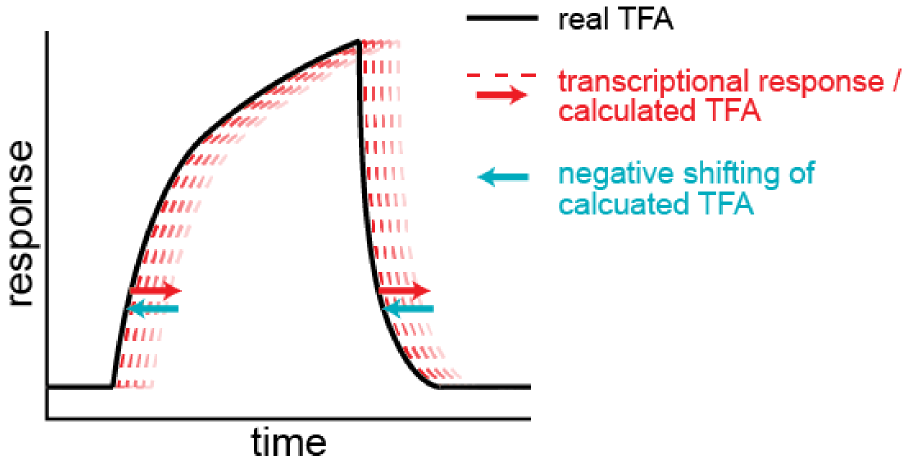

Supplementary Figure 10. Schematic of the potential time lag between real and calculated transcription factor activity (TFA). Real TFA follows immediately the change in effector metabolite level (black). Because of a delay in gene expression, the actual transcriptional response has a negative time-lag (red). Therefore we allow a negative time-lag for TFA in the correlation analysis with metabolites (blue arrows).
