# Supplementary material for: Systematic identification of metabolites controlling gene expression in E. coli
Source: Nat Commun. 2019 Oct 2;10:4463. doi: 10.1038/s41467-019-12474-1 (PMC6775132; doi:10.1038/s41467-019-12474-1)
Supplement: Supplementary file 3 — Description of Additional Supplementary Files [file 41467_2019_12474_MOESM3_ESM.pdf]

Description of Additional Supplementary Files

File Name: Supplementary Data 1
Description: Fold change and intracellular concentrations (µM) of measured metabolites in *E. coli* BW25113 in the switch from growth to starvation and back to growth. Fold change is calculated to the first time point t0. Compound information contains KEGG, metabolite name, BIGG identifier and the corresponding subsystem.

File Name: Supplementary Data 2
Description: Expression of genes shown as TPM (Transcripts Per kilobase Million) of *E. coli* BW25113 in the switch from growth to starvation and back to growth.

File Name: Supplementary Data 3
Description: Process time in h, optical density at 600 nm (OD600), growth rate (µ), reactor volume Vr in Liter and the corresponding metabolite and transcript samples during the shift of starvation and growth. Samples for metabolomics were collected at 35 time points; samples for transcriptomics were collected at 29 time points.

File Name: Supplementary Data 4
Description: Process data including process time (h), agitation (RPM), dissolved oxygen DO (%), gas flow inlet (sL*min^-1), pH, temperature (°C), gas flow outlet (sL*h^-1), oxygen concentration O2 (%), carbon dioxide concentration CO2 (%), oxygen uptake rate OUR (mmol*h^-1), carbon production rate CPR (mmol*h^-1), specific oxygen uptake rate qO2 (mmol*h^-1*g^-1) and specific carbon production rate qCO2 (mmol*h^-1*g^-1) during the shift of starvation and growth.

File Name: Supplementary Data 5
Description: Transcription factor activity (log 10) calculated by Network Component Analysis and the 95% confidence interval of 100 calulations with randomised starting points.

File Name: Supplementary Data 6
Description: Correlation coefficients of 123 metabolites and 125 transcription factor activities using Hill-type parameters

File Name: Supplementary Data 7
Description: 513 correlation coefficients of metabolite- transcription factor pairs that fulfill the distance filter.

File Name: Supplementary Data 8
Description: Connectivity matrix of the transcriptional regulatory network between all transcriptional regulators and genes in *E. coli*. Adapted from RegulonDB and EcoCyc. 1 indicates activation; -1 indicates inhibition by the transcriptional regulator.

File Name: Supplementary Data 9
Description: Gene expression input (log10) for Network Component Analysis.

File Name: Supplementary Data 10
Description: Reporting standards for metabolit measurement
